# HLA RNA Sequencing With Unique Molecular Identifiers Reveals High Allele-Specific Variability in mRNA Expression

**DOI:** 10.3389/fimmu.2021.629059

**Published:** 2021-02-25

**Authors:** Tiira Johansson, Dawit A. Yohannes, Satu Koskela, Jukka Partanen, Päivi Saavalainen

**Affiliations:** ^1^ Research Programs Unit, Translational Immunology Program, University of Helsinki, Helsinki, Finland; ^2^ Research and Development, Finnish Red Cross Blood Service, Helsinki, Finland

**Keywords:** HLA, allele-specific expression, RNA sequencing, unique molecular identifiers, gene expression

## Abstract

The HLA gene complex is the most important single genetic factor in susceptibility to most diseases with autoimmune or autoinflammatory origin and in transplantation matching. Most studies have focused on the vast allelic variation in these genes; only a few studies have explored differences in the expression levels of HLA alleles. In this study, we quantified mRNA expression levels of HLA class I and II genes from peripheral blood samples of 50 healthy individuals. The gene- and allele-specific mRNA expression was assessed using unique molecular identifiers, which enabled PCR bias removal and calculation of the number of original mRNA transcripts. We identified differences in mRNA expression between different HLA genes and alleles. Our results suggest that HLA alleles are differentially expressed and these differences in expression levels are quantifiable using RNA sequencing technology. Our method provides novel insights into HLA research, and it can be applied to quantify expression differences of HLA alleles in various tissues and to evaluate the role of this type of variation in transplantation matching and susceptibility to autoimmune diseases.

## Introduction

The highly polymorphic human leukocyte antigens (HLA) are crucial in presentation of self, non-self and tumor antigens to T cells, and play an important part in autoimmunity and infection responses, as well as in organ and hematopoietic stem cell transplantation (HSCT). In the thymus and bone marrow, the HLA molecules presenting self-derived peptides to maturing T- and B-cells induce the central tolerance. The classical HLA genes are divided into two classes. HLA class I genes including *HLA-A, HLA-B*, and *HLA-C* are expressed on the surface of all nucleated cells, whereas the expression of class II genes; *HLA-DR, HLA-DQ*, and *HLA-DP* is restricted to professional antigen presenting cells (1, 2). Recent studies have reported varying expression levels of HLA alleles based on the quantitative polymerase chain reaction (qPCR) (3–9) and the mean fluorescence intensity (MFI) (10, 11). The differential expression of HLA genes and alleles has been associated with immunologically mediated diseases, such as Crohn’s disease (7) and HIV (11, 12), follicular lymphoma (9), lung cancer (13), ovarian cancer (14, 15), and the outcome of HSCT through the increased risk of graft versus host disease (GvHD) (5, 10, 16). In addition to allele-specific differences in the constitutive expression, a recent study also found HLA allele-specific expression to vary during T cell activation (17). These differences in HLA expression may partly explain the susceptibility to autoimmune diseases, tumor invasion, and infections. In HSCT, the incompatibilities between the donor and the recipient have made the expression differences of HLA molecules an interesting target for finding permissive mismatches. Although currently only qualitative HLA typing is considered in donor selection, techniques based on RNA sequencing (RNA-Seq) can be used to determine differences in HLA expression that may influence the outcome of the transplantation.

In the past years, next generation sequencing (NGS) has enabled the rapid development of several novel high-throughput HLA typing methods on different sequencing platforms (18–25). Unlike genomic DNA based applications, RNA-Seq provides comprehensive gene expression information in addition to HLA allele calling. Several existing tools (26–29) have been developed to perform HLA typing from short RNA-Seq reads using whole transcriptome data. In addition, multiple existing computational tools enable HLA expression quantification from RNA-Seq data. A tailored gene quantification pipeline was applied to publicly available RNA-Seq datasets to obtain expression estimates from different cancer cell lines (30) and 56 normal tissues and cell types (31). A different computational pipeline provided both gene-level and allele-level expression estimates (32), and an alternate reference was used to generate gene-level and haplotype-level estimates of transcript abundance (33). Additionally, a method with capture probes together with RNA-Seq was used to quantify HLA allele-specific expression from 161 peripheral blood mononuclear cells (PBMCs) and 48 umbilical cord blood cells (34). In comparison to qPCR and fluorescence-based methods, RNA-Seq is less laborious and time-consuming. However, it has its own challenges. With over 27,000 different HLA alleles reported by the IPD IMGT/HLA database (Release 3.41.2, https://www.ebi.ac.uk/ipd/imgt/hla/), a precise identification of HLA alleles from short-read NGS data is challenging. The highly polymorphic and homologous nature of HLA genes often leads to ambiguous results in the allele assignment. Additionally, in HLA expression quantification short RNA-Seq reads may cause bias by mapping to several HLA alleles or even several genes (35). This may lead to a situation where many reads aligning to multiple alleles are excluded from the analysis. There are some ways to overcome this problem. One option to avoid multi-mapping reads is to use longer sequencing reads to cover more polymorphic positions between different alleles. Longer reads are unlikely to align multiple positions and hence make the alignment more accurate. Another is to use a sample-specific HLA reference in the expression quantification step. A reference, which contains only the known alleles will drastically reduce the number of alleles where a read can align. In addition to RNA-Seq reads mapping to multiple locations, PCR duplicates can also cause bias in the HLA expression quantification. Several RNA-Seq methods have a PCR amplification step in the library preparation protocol to expand the starting material. The problem is the potential differences in the amplification efficacy, which may lead to overrepresentation of some molecules (36, 37). By incorporating unique molecular identifiers (UMIs) in the library preparation as molecular barcodes, it is possible to distinguish PCR duplicates derived from a single molecule in the data analysis step (38). The number of UMI combinations must be high enough for all molecules in the starting pool to receive a different UMI (39). Both five and ten nucleotides long UMIs have proven to be efficient to correct the PCR-induced artifacts, and to accurately count only the original transcripts from RNA-Seq data (38, 40).

Here, we describe a highly multiplexed RNA-based sequencing method using UMIs to quantify HLA gene- and allele-specific expression from PBMCs of 50 healthy blood donors. For accurate, high-throughput quantification of the expression levels of HLA genes and alleles, we developed a bioinformatics pipeline, written in R, based on counting of UMIs to distinguish original transcripts from PCR copies.

## Materials and Methods

### Samples and RNA Extraction

This study collected 50 healthy blood donor buffy coat samples with a negative HIV, HBsAg, and HCV status, which underwent an isolation of pheripheral blood mononuclear cells using Ficoll-Paque**™** Plus (GE Healthcare), Dulbecco**’**s Phosphate Buffered Saline DPBS CTS**™** (Gibco life technologies), Fetal Bovine Serum FBS (Sigma) and SepMate™-50 tubes following the manufacturer**’**s protocol (Stemcell Technologies). The use of anonymized PBMCs from blood donors was conducted in accordance with the rules of the Finnish Supervisory Authority for Welfare and Health (Valvira). Cell count was measured from a mix of 50 µl of cell suspension in DPBS with 2% FBS, 50 µl of Reagent A100 lysis buffer, and 50 µl of Reagent B stabilizing buffer using a NucleoCassette and a NucleoCounter**^®^** NC-100™ (all chemometec). Total RNA was isolated from fresh PBMC samples containing 1–10 × 10^6^ cells using RNeasy Mini kit and Rnase-Free DNAse Set (both Qiagen) within 2h after PBMC isolation. RNA samples were quantified and their purity was assessed with the Qubit™ RNA High Sensitivity Assay Kit in Qubit**^®^** 2.0 fluorometer (ThermoScientific). The RNA quality was checked using an RNA 6000 Pico Kit (Agilent Genomics) in a 2100 Bioanalyzer (Agilent Genomics) to obtain an RNA Integrity Number (RIN) score.

### Reverse Transcription by Template Switching and Target Amplification

We used an adaptation of the STRT method to generate full length complementary DNA (cDNA) molecules from RNA transcripts (41). Briefly, the poly-A hybridization to the first strand cDNA synthesis primer was performed in a 96-well plate in a T100™ Thermal Cycler (Biorad) with 3 min at 72°C with 25 ng of RNA, 1% Triton™ X-100 (Sigma), 20 µM of STRT-V3-T30-VN oligo, 100 µM of DTT (invitrogen, life technologies, Thermo Fisher), 10 mM dNTP (Bioline), 4 U of Recombinant RNase Inhibitor (Takara Clontech), 1:1,000 The Ambion^®^ ERCC RNA Spike-In Control Mix (life technologies, Thermo Fisher) in a total volume of 3 µl. All oligos were from Integrated DNA Technologies and are listed in **Table S1**. Reverse transcription of the whole transcriptome was performed adding 3.7 µl of the RT mix containing 5× SuperScript first strand buffer (invitrogen by Thermo Fisher Scientific), 1 M MgCl_2_ (Sigma), 5 M Betaine solution (Sigma), 134 U of SuperScript ^®^ II Reverse Transcriptase (invitrogen by Thermo Fisher Scientific), 40 µM RNA-TSO 10bp UMI, 5.6 U of Recombinant RNase Inhibitor immediately to each reaction. To complete the reverse transcription and the template switching, the plate was incubated 90 min at 42°C followed by 10 min at 72°C. In this reaction, every transcript receives a unique distinct barcode. After RT the cDNA was further amplified with 2× KAPA HiFi HotStart ReadyMix (Kapa Biosystems), 10 µM ImSTRT-TSO-PCR with a thermal profile consisted of an initial denaturation of 3 min at 95°C followed by 20 cycles of 20 s at 95°C, 15 s 55°C, 30 s at 72 and one cycle of final elongation of 1 min at 72°C in a final volume of 50 µl. Qubit™ dsDNA High Sensitivity Assay Kit (Thermo Fisher Scientific) was used to measure the concentration of all cDNA samples. The 3’ fragments of the cDNA were released in a restriction reaction using SalI-HH (New England Biolabs) according to the manufacturer’s protocol. The concentration of DNA was measured using Qubit™ dsDNA High Sensitivity Assay Kit and DNA integrity and the size distribution were assessed with High Sensitivity DNA Kit (Agilent Genomics). For HLA target enrichment one TSO-specific universal forward primer and eight gene-specific reverse primers with universal tails for amplicon sequencing were used to amplify exons 1 to 8 in class I genes *HLA-A, -B*, and *-C* or exons 1 to 5 in class II genes *HLA-DRA, -DRB1, -DPA1, -DPB1, -DQA1* and -*DQB1*. *HLA-A, -B*, and *-C* had one common primer. All seven gene-specific primers were designed to fall within a non-polymorphic region using the known sequence diversity, as described in the international ImMunoGeneTics IMGT/HLA database (http://www.ebi.ac.uk/imgt/hla/). The amplification was performed in 96-well plates with 3 µl of template cDNA, 10× Advantage 2 PCR buffer, 50× Advantage^®^ 2 Polymerase Mix (Takara, Clontech), 10 mM dNTP (Bioline), 10 µM TSO forward primer and one of the seven HLA gene-specific reverse primers in a total volume of 15 µl. The PCR reaction consisted of an initial denaturation of 30 s at 98°C following three cycles of 10 s at 98°C, 30 s at 55°C, 30 s at 72°C and 27 cycles of 10 s at 98°C, 30 s at 71°C, 30 s at 72°C and final elongation of 5 min at 72°C. To confirm the amplicon lengths and non-specific amplification, 4 samples were selected from each plate with the amplification performed using different gene-specific primer. These samples were run on a 2% agarose gel (Bioline) with 10× BlueJuice™ loading dye (invitrogen by Thermo Fisher Scientific) in 0.5× TBE (Thermo Fisher Scientific) with the GelGreen™ (Biotium) and visualized using the Quick-Load 1kb DNA Ladder (New England Biolabs). DNA of the PCR amplicons was quantified with the Qubit™ dsDNA High Sensitivity Assay Kit and the fragment sizes analyzed with Agilent’s High Sensitivity DNA Kit.

### Illumina Library Preparation and Sequencing

For Illumina sequencing, all genes of 50 HLA amplicons were multiplexed per sample. 50 cDNA and 50 HLA amplicon libraries were prepared using the Nextera XT DNA Library Preparation Kit (Illumina). For an optimal insert size and a library concentration 600 pg of each cDNA and PCR amplicon sample was tagmented for 5 min at 55°C using 5 µl of Nextera’s Tagment DNA Buffer, 0.25 µl of Nextera’s Amplicon Tagment Mix in a final volume of 10 µl. The transposone was inactivated with 2.5 µl of Nextera’s Neutralize Tagment Buffer for 5 min at room temperature. The dual indexing and adapter ligation took place in a PCR reaction with 7.5 µl of Nextera PCR Master Mix, 4 µl of nuclease-free water and 10 µM of i5 custom oligo and 10 µM of Nextera i7 N7XX oligo using a limited-cycle PCR program: an initial denaturation 30 s at 95°C following 12 cycles of 10 s at 95°C, 30 s at 55°C, 30s at 72°C with a final elongation step of 5 min at 72°C. After the amplification all 50 cDNA and HLA amplicons samples were pooled together into two separate pools, one cDNA and one HLA amplicon pool. These two pools were then purified twice using the Agencourt AMPure XP beads according to the manufacturer’s instructions first with 0.6× beads:DNA ratio and then with 1× beads:DNA ratio and eluted in 30 µl. Qubit™ dsDNA High Sensitivity Assay Kit was used to quantify DNA and HT DNA HiSens Reagent kit and DNA Extended Range LabChip in LabChip GXII Touch HT (all PerkinElmer) to assess the size distribution of the libraries. A double size selection was performed with the Agencourt AMPure XP beads according to the manufacturer’s instructions to remove fragments over 1,000 bp (0.8× beads:DNA ratio) and under 300 bp (0.6× beads:DNA ratio). Prior to sequencing the DNA concentration was assessed with Qubit™ dsDNA High Sensitivity Assay Kit HT DNA HiSens Reagent kit and the library size verified with HT DNA HiSens Reagent kit. The two pooled and barcoded libraries were denaturated with 0.2 M NaOH and diluted in the HT1 buffer to obtain a final library concentration of 20 pM in 0.95:0.05 cDNA : HLA amplicon ratio. The libraries were sequenced by using Illumina Nextseq sequencer with 300 cycles (NextSeq 500/550 v2) kits generating 100 bp (read 1) and 200 bp (read 2) reads.

### Data Analysis

Paired-end reads from cDNA and HLA amplicon libraries in fastq format underwent an UMI extraction using the UMI-tools (v0.5.11) (42), a quality control step using FastQC (43), and were quality trimmed using trimmomatic (v0.35). Processed cDNA library reads were aligned using HISAT2 (v2.1.0) (44) to the human genome (GRCh38) and assigned to genes according to the UMI-tools pipeline using featureCounts tool from the subread package (v1.5.3) (45). Samtools (v1.4) were used to sort and index BAM files and UMI-tools count tool to count the number of unique UMIs per gene. The set of 50 count files were then merged into a single count table using the Define NGS experiment tool in Chipster (v3.12.2) (46).

To quantify HLA expression from RNA-Seq reads, we implemented the strategy of assessing allele-specific expression by aligning reads, using LAST (47), to sample-specific personalized HLA references extracted from the IPD IMGT/HLA reference database. The personalized HLA references, which contain the reference sequences of all the HLA alleles specific for a sample, were built based on the information from prior HLA typing. To retain all reads that originate from the HLA region, alignment results from separate alignment of R1 reads, R2 reads, and paired-end alignment (using last-pair-probs) were combined. We selected LAST to allow optimal alignment with a relatively “permissive” initial step to collect all HLA originating reads. By changing the default parameters, we opted LAST, and performed the more strict read discrimination tasks in the latter steps. We used LAST with the parameters -s 2 -T 0 -l 50 -a 100 -Q 1 -i1, with a long minimum initial seed length of 50 to enforce strict long initial seed matching (-l 50, the default last setting for this parameter was 1), and a very prohibitive cost for opening gaps to decrease the chance of any read with some similarity aligning to the references (-a 100, default 7). This first alignment step filtered out reads that do not map to any of the alleles in the personalized HLA reference for a sample. The set of reads that aligned to any of the alleles in the personalized reference were retained, and their UMIs and aligned portions along with their base qualities were extracted from the LAST output file (in MAF file format). UMIs were extracted from the read names of the aligned reads. These aligned reads were processed further as described next.

The HLA aligning reads for a sample were first grouped by their UMIs, and each UMI (i.e, reads having the same UMI or UMIs that differ by at most 1 nucleotide, which are assumed to originate from the same transcript) were then evaluated separately for assignment to HLA alleles. The total number of UMI assignments or counts for each allele represent the estimated allele-specific expression after UMI deduplication. Prior to assigning the UMIs to HLA alleles, the key polymorphic sites between the alleles in the personalized HLA reference sequences were identified. First, by performing multiple alignment of the sequences (using msa R package) (48), and then obtaining the positions with high diversity (Shannon entropy index > 0.5) from the consensus matrix of the sequences (generated using Biostrings v2.46.0 and ShortRead R packages) (49, 50). The corresponding bases at the polymorphic sites were identified for all alleles in the personalized reference. To assign each UMI to an HLA allele, the result from the LAST alignment was processed further. UMIs that have reads aligning to only one specific allele were counted to that allele so that each UMI is counted just once. For each UMI that has reads mapping to multiple alleles, the aligned portions of the reads from LAST are re-aligned to the personalized HLA reference sequences using overlap (end-to-end) alignment (pairwiseAlignment function of Biostrings R package). Then, a Bayesian based statistical model was used to assign the UMIs to one of the reference alleles as follows. The UMI’s likelihood of originating from each of the reference alleles, *P*(*U|A*), was calculated based on how well the reads of the UMI, from the end-to-end alignment, match the corresponding bases of each reference allele at the key polymorphic sites with:

$P\left(U|A_{i}\right)=\frac{1}{n}\sum_{k=1}^{n}p(b_{k}|A_{ik})$ where, n is number of key polymorphic sites covered by reads of the UMI, *b_k_* is the observed base at the key site k in U, *A_ik_* is the reference base at key site k for the reference HLA allele i, and U is the UMI. The probability of the observed base at each key position given an HLA allele was calculated as:

$p(b_{k}|A_{ik})=\{\frac{1-e,\;if\;b_{k}=A_{ik}}{e/3,\;if\;b_{k}\neq A_{ik}}$, where e is base-specific sequence error given by e=10^(-q/10) and q is the sequencing quality of the base in phred score.

The likelihood was calculated as the sum of the probabilities of the observed bases, p(*b*|*A*), assuming each covered key site (by the reads of the UMI) contributes 1/n of the total likelihood, which is considered to be an aggregate of only the key polymorphic sites covered by the reads of the UMI, n. A likelihood close to 1 suggests strong match between the UMI and the reference allele. UMIs were then assigned to the HLA reference allele with the highest likelihood, and counted once to that allele (i.e, are de-duplicated upon counting). The R script of this pipeline (HLAXPress), which implements the allele-specific estimation analysis described above can be found at https://github.com/dyohanne/HLAXPress.

After HLA expression quantification, a normalization of Illumina cDNA and HLA amplicon reads took part. First, HLA gene-specific counts resulting from the alignment of cDNA reads to the human genome were removed and replaced in the merged count table with HLA allele-specific UMI counts derived from cDNA reads after the HLAXPress pipeline. Second, read counts were normalized to counts per million (CPM) using the cpm tool from the limma package (v3.30.13) (51). For Illumina HLA amplicon libraries, UMIs of each allele were normalized first by calculating unique UMI proportions between alleles out of the total number of UMIs per sample. Then, for each individual these proportions were multiplied by the total number of CPM-normalized unique UMIs of all HLA alleles in cDNA library.

### Statistical Analysis

All statistical analyses were performed using GraphPad Prism v8.4.2 (GraphPad Software). Statistical significance of gene-, allotype, and haplotype-level expression were analyzed using the non-parametric Kruskal-Wallis test or the Mann-Whitney U test. In the gene-level expression analysis, the Kruskal-Wallis test was followed by the Dunn’s multiple comparisons test. The Spearman correlation coefficients were applied in the comparison of allelic ratios between the datasets. In all tests, p-values < 0.05 were considered statistically significant.

### Software Availability

HLAXPress is freely available at https://github.com/dyohanne/HLAXPress.

## Results

### Method Development

**Figure 1:**
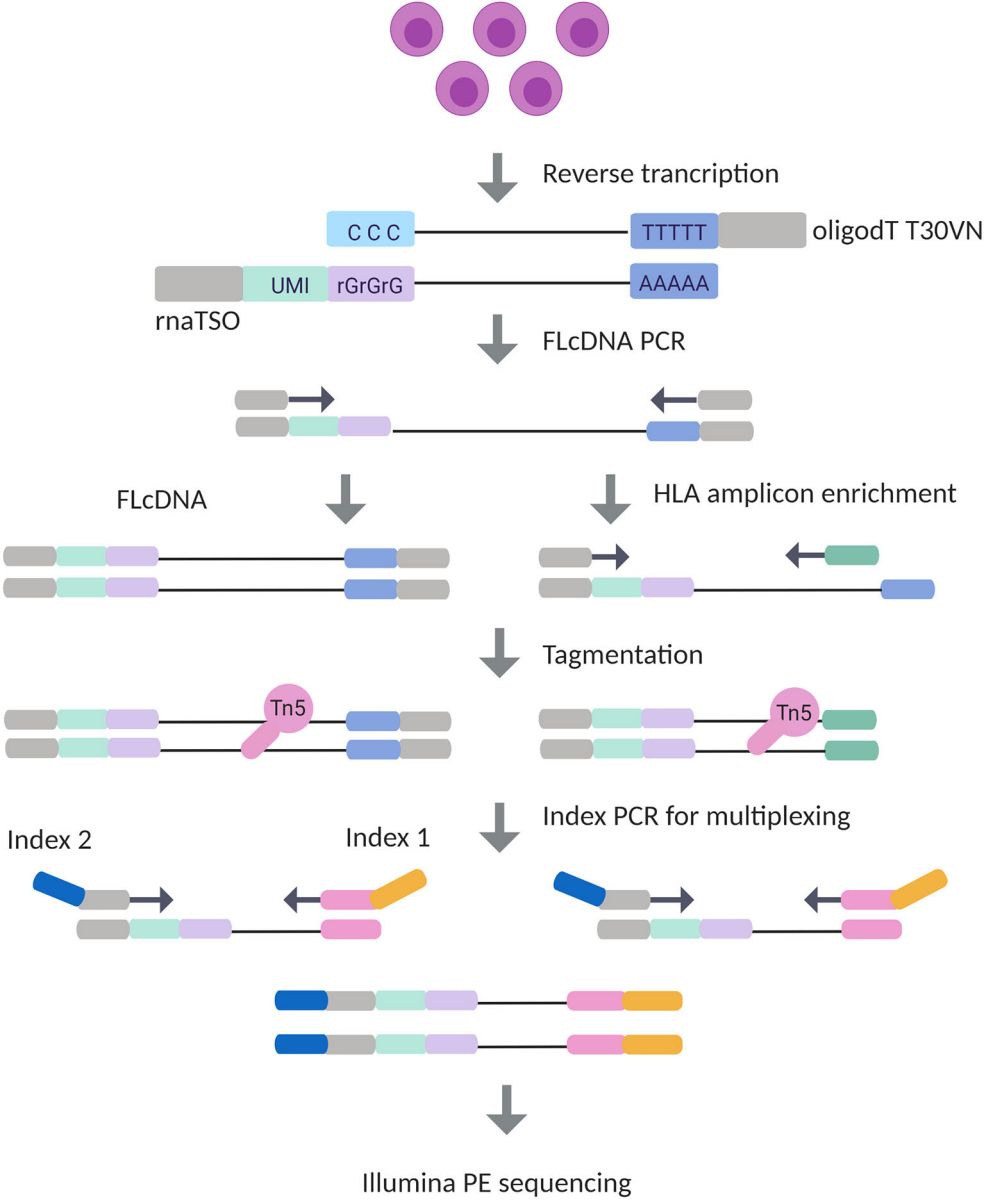
Experimental design of Illumina HLA RNA-Seq. In the library preparation process, mRNA is first transcribed into complementary DNA (cDNA) with simultaneous integration of 10 bp unique molecular identifier (UMI) in RNA template switching oligo (rnaTSO) and further amplified. The full-length cDNA (FLcDNA) is then divided and processed in parallel in Illumina’s cDNA and amplicon library preparation protocols. The amplicon library preparation included an enrichment of HLA genes using gene-specific primers. Lastly, the full-length cDNA and HLA amplicons are tagmented, and sample-specific barcodes are added for multiplexing. The method results 5’ end library molecules suitable for Illumina paired-end sequencing.

To determine HLA gene- and allele-specific expression using UMIs, we developed an RNA-Seq protocol (**Figure 1**) based on the STRT method (40). Our method included an incorporation of 10 bp UMI to the 5’ end of RNA transcripts using a template switching oligo (TSO) in the first strand synthesis followed by an amplification of the full-length complementary DNA (cDNA). A pool of 10 bp UMI equaled to 1,048,576 unique barcodes, which were implemented to improve the PCR duplicate removal. To compare the HLA expression using the full transcriptome and targeted HLA genes, the full-length cDNA library was split, and processed in parallel in HLA cDNA and HLA amplicon library preparation protocols. Nine gene-specific primers were designed as reverse primers to enrich HLA genes together with a TSO primer from full-length cDNA. Both libraries underwent tagmentation, dual-indexing using PCR, and sequencing in a single run on Illumina’s Nextseq 500 cycles kit.

#### Sequence Read Analysis and HLA Genotyping

Both, Illumina cDNA and amplicon library reads underwent an UMI extraction (42), a quality overview step using FastQC (43), and preprocessing using Trimmomatic (52). After trimming, 55,100 to 1,583,520 reads for the cDNA library and 6,200 to 44,110 reads for the amplicon library remained for the mRNA expression quantification. HLA typing using an ensemble method (53) and Luminex SSOP-PCR (Method S1) assigned 52 different HLA class I alleles and 56 different HLA class II alleles at 2-field level (**Table S2**). The number of different alleles per gene were 14 (*HLA-A*), 24 (*HLA-B*), 14 (*HLA-C*), 2 (*HLA-DRA*), 18 (*HLA-DRB1*), 4 (*HLA-DPA1*), 10 (*HLA-DPB1*), 11 (*HLA-DQA1*), and 11 (*HLA-DQB1*), and the heterozygosity rates were 62%, 94%, 92%, 16%, 90%, 24%, 78%, 82%, and 89%, respectively (**Table 1**). Based on the HLA genotyping information we built personalized sample-specific HLA references at 2-field resolution level for all 50 individuals.

**Table 1:** The number of different alleles and the heterozygosity rates of nine human leukocyte antigen (HLA) genes of 50 individuals.

HLA gene	Number of different alleles	Heterozygosity rate (%)
**HLA-A**	14	62
**HLA-B**	24	94
**HLA-C**	14	92
**HLA-DRA**	2	16
**HLA-DRB1**	18	90
**HLA-DPA1**	4	24
**HLA-DPB1**	10	78
**HLA-DQA1**	11	82
**HLA-DQB1**	11	89

#### Comparison of HLA Expression Quantification Between Two Illumina Datasets

**Figure 2:**
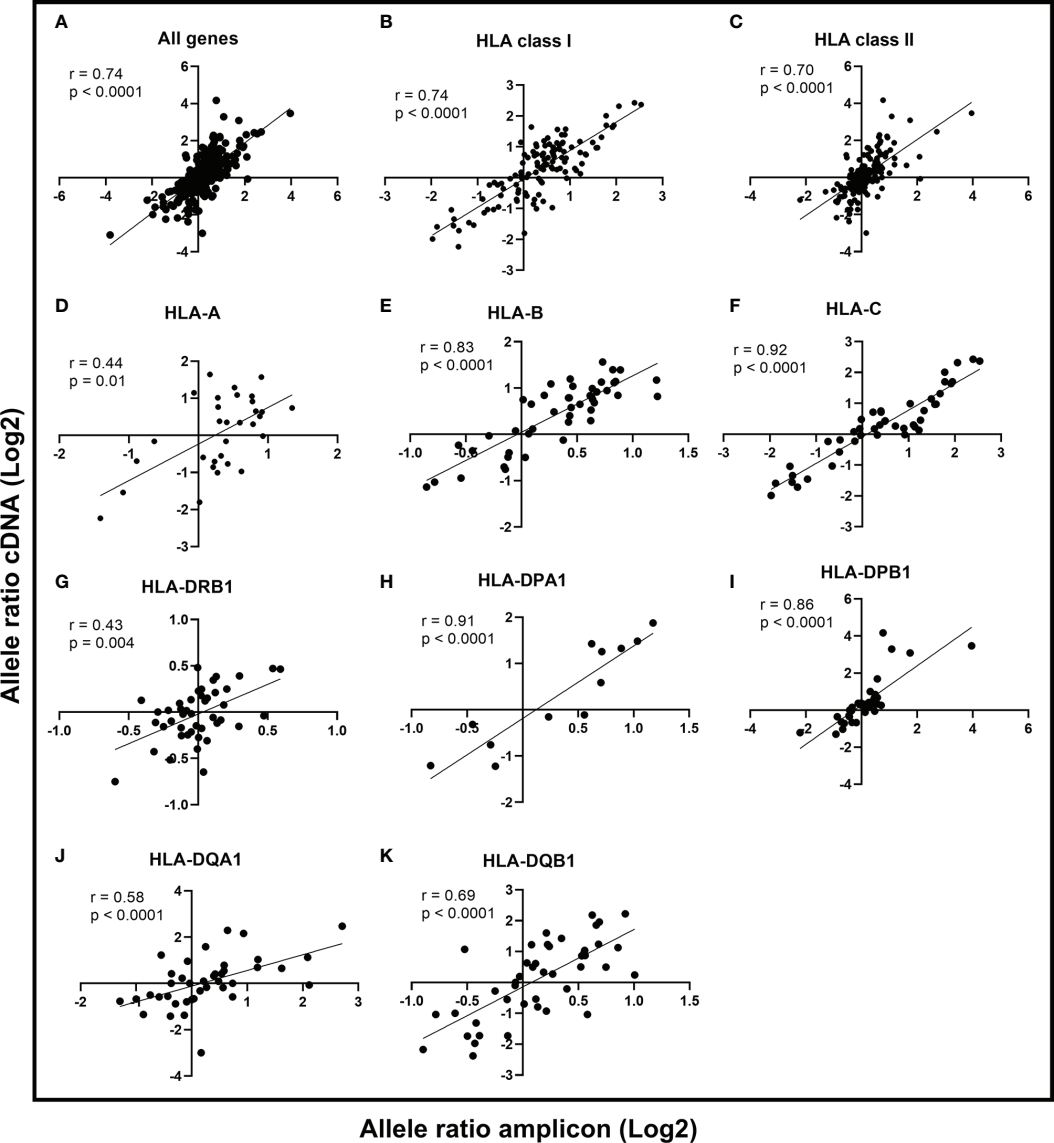
Comparison of allele ratios between Illumina cDNA and Illumina amplicon datasets. The allele expression ratio was calculated for each heterozygous allele pair in the two datasets and a non-parametric Spearman’s rank correlation was used to compare the allele-level expression between cDNA and amplicon data. The line indicates the linear regression. The Spearman correlation coefficient is given for all genes **(A)**, HLA class I **(B)**, HLA class II **(C)**, and for genes HLA-A **(D)**, HLA-B **(E)**, HLA-C **(F)**, HLA-DRB1 **(G)**, HLA-DPA1 **(H)**, HLA-DPB1 **(I)**, HLA-DQA1 **(J)**, and HLA-DQB1 **(K)**.

In the comparison of the correlation between Illumina cDNA and Illumina amplicon data, we calculated the allele-to-allele ratio from unnormalized unique UMIs for each heterozygous allele pair among all 50 samples. The correlation of the allele ratios (log2) between Illumina cDNA and Illumina amplicon data was strong (r = 0.74, p < 0.0001; Spearman rank correlation) with all nine HLA genes included in the analysis (**Figure 2A**). This suggested that both datasets alone were able to identify the expression difference between the two alleles. The correlation of HLA class I genes and class II genes were r = 0.74 (p < 0.0001) and r = 0.70 (p < 0.0001), respectively (**Figures 2B, C**). The gene-level comparison of the allele ratios revealed that the strongest correlation were in *HLA-B* (r = 0.83, p < 0.0001), *HLA-C* (r = 0.92, p < 0.0001), *HLA-DPA1* (r = 0.91, p < 0.0001), and *HLA-DPB1* (r = 0.86, p < 0.0001) (**Figures 2E, F, H, I**). *HLA-DQA1* and *HLA-DQB1* showed a moderate correlation with r = 0.58 (p < 0.0001) and r = 0.69 (p < 0.0001), respectively (**Figures 2J, K**). The weakest correlations were in *HLA-A* (r = 0.44, p < 0.01) and *HLA-DRB1* (r = 0.43, p = 0.004) (**Figures 2D, G**).

#### Method Validation

To explore the accuracy our HLA RNA-Seq method, we determined the gene- and allele-level mRNA expression of *HLA-C* of five samples using qPCR (Method S2). The gene-level comparison showed a high correlation (r = 0.9, p < 0.08) between qPCR and our HLA RNA-Seq method (Method S2: **Figure S2**). For the allele-level comparison, we chose four samples with C*06:02~C*07:01 allele pairs and one sample with C*04:01~C*07:01. Both HLA RNA-Seq and qPCR showed similar expression patterns at allele-level mRNA expression in C*06:02~C*07:01 allele pairs (**Method S2**: **Figure S3**). However, in C*04:01~C*07:01, the expression difference between the alleles was greater in qPCR than in RNA-Seq. This resulted from a higher mRNA expression of allele C*04:01 in qPCR. Conversely, the expression of C*07:01 was similar in both methods. We also compared the gene-level mRNA expression between our method and the previously published capture RNA-Seq method (34). **Figure S4** shows a high correlation (r = 0.9, p < 0.0009) between these two methods indicating that our HLA RNA-Seq method can accurately quantify HLA gene-specific expression from RNA-Seq data using UMIs. We acknowledge that method-related biases and experimental factors are possible causes of variation in results between qPCR and RNA-Seq. Hence, further studies in the future with several alleles and genes are required to evaluate the comparability between these two methods.

### HLA Gene-Specific Expression

**Figure 3:**
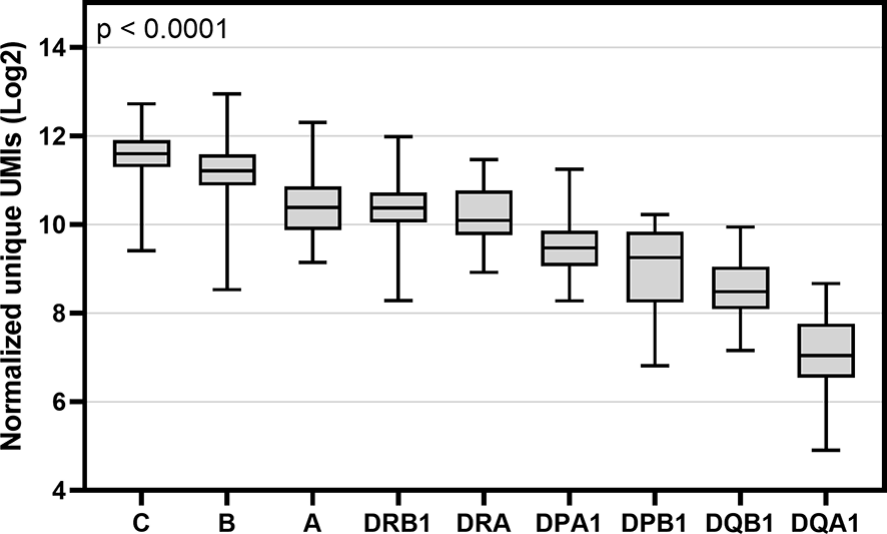
Box and whisker plot of gene-specific expression of nine HLA genes of 50 individuals. Y-axis indicates the sum of normalized unique UMIs (Log2) of two alleles of cDNA data and X-axis indicates the names of nine HLA genes. Boxes indicate the lower quartile, median and upper quartile. Data were compared using a Kruskal-Wallis test.

An in-house software tool, HLAXPress, together with personalized allele references counted the number of unique UMIs representing the mRNA expression of HLA (**Tables S3** and **S4**). HLAXPress outputs a report with the UMIs per allele. For this reason, to characterize the gene-specific expression, the allele-specific UMI counts were first normalized to library size using the CPM method, and then summed together to get the gene-specific UMI counts. **Figure 3** shows the expression profiles of nine HLA genes of 50 individuals using the cDNA data. Among the nine HLA genes, there was a significant statistical difference (Kruskal-Wallis test p < 0.0001) in the gene-level expression comparison. The pairwise comparison of the gene-specific expression between nine HLA genes using a Dunn’s test is shown in **Table S5**. Among the class I and class II genes, *HLA-B* and *-C* were expressed at the highest levels. The average level of *HLA-A* gene expression was about 2 times lower compared to the expression of *HLA-C*. In the HLA class II *HLA-DRA* and *-DRB1* genes were expressed at the highest levels following *-DPA1* and *-DPB1*. *HLA-DQA1* and *-DQB1* were expressed at the lowest levels.

### HLA Allotype Expression

**Figure 4:**
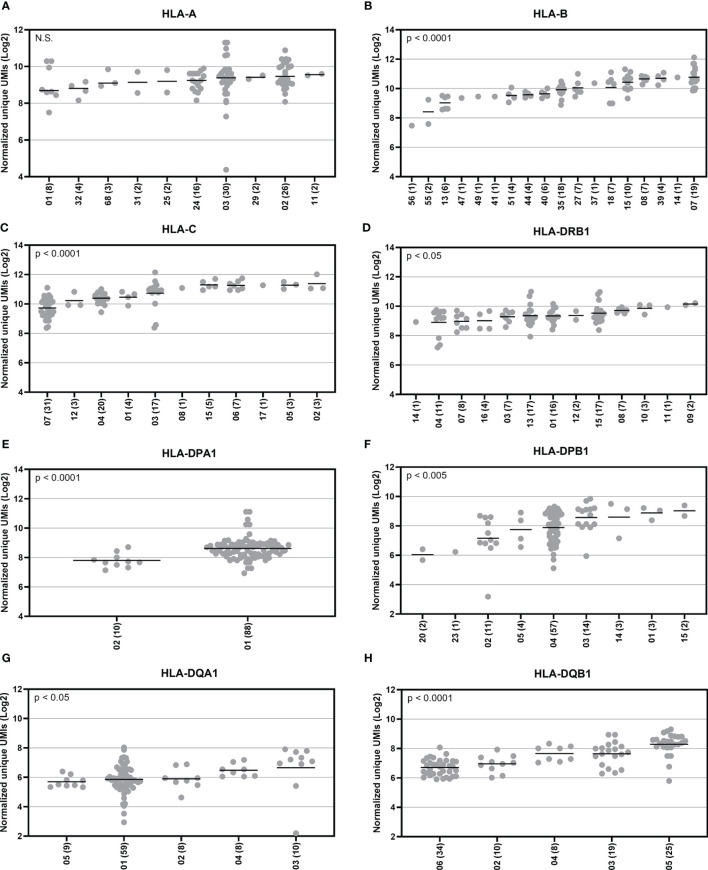
Allotype expression of HLA class I and class II genes of 50 individuals. Allele-level UMIs of eight HLA genes **(A–H)** were normalized, grouped to allelic lineages, and plotted according to different lineages in Illumina cDNA data. Y-axis indicates the normalized unique UMIs (Log2) and X-axis indicates the names of the allelic lineages. Each dot refers to a UMI value and a solid bar indicates the median expression of the lineage. The number of samples are shown within parentheses. A Kruskal-Wallis test compared the ranks between the allelic lineages.

**Figure 5:**
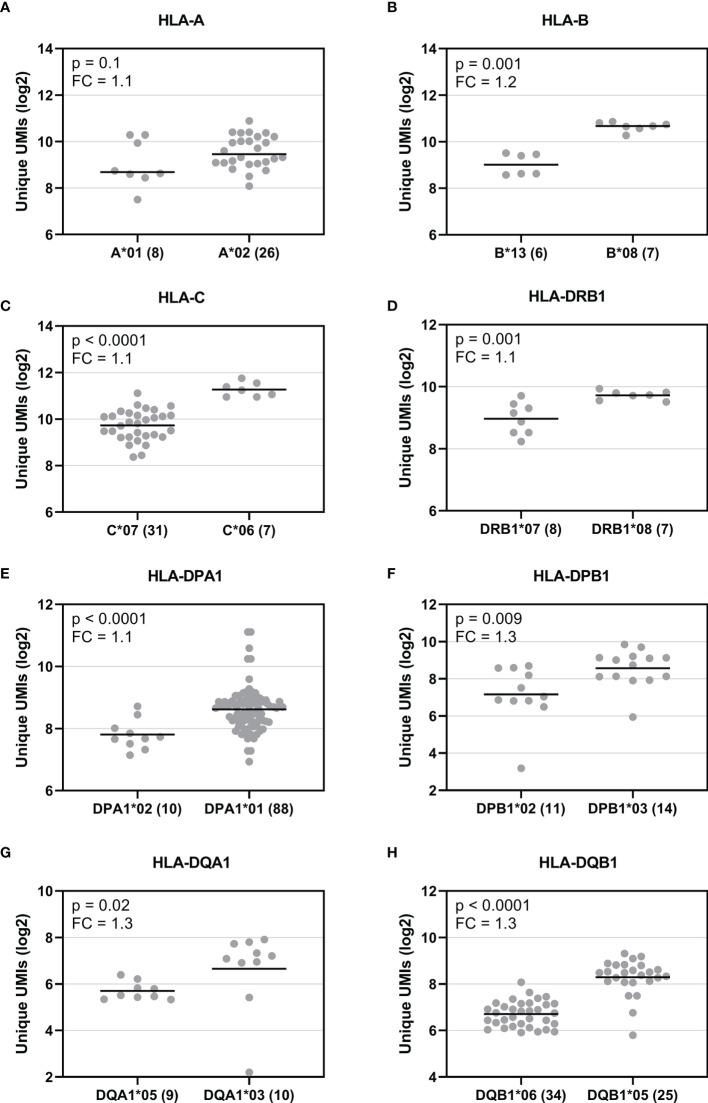
Comparison of the mRNA expression between the highest and lowest expressed allelic lineage of eight HLA genes. Y-axis indicates the normalized UMIs (Log2) of cDNA data and X-axis indicates the names of the selected allelic lineages of eight HLA genes **(A–H)**. Each dot indicates a UMI value and a solid bar indicates the median expression of the lineage. The number inside the parenthesis indicates the number of samples. The expression between the allelic lineages was compared using a Mann-Whitney U test.

To explore HLA allele-specific expression, the normalized unique UMI values of different alleles were first grouped by combining serologically equivalent alleles into HLA allelic lineages due to the small sample size per allele. **Figure 4** shows the expression profiles of allelic lineages of eight HLA genes of 50 individuals. *HLA-DRA* formed only one allelic lineage and hence was excluded from the analysis. The results suggested that despite a high variation in allele-level expression between individuals, the differences in the mRNA expression between allelic lineages are significant. A Kruskall-Wallis test showed a significant statistical difference in expression of allelic lineages among seven HLA genes: *HLA-B, -C, -DRB1, -DPA1, -DPB1, -DQA1*, and *-DQB1* (**Figures 4B–H**). However, in *HLA-A*, the mRNA expression was not significantly different between allelic lineages (**Figure 4A**). We also investigated the magnitude of the differential allele-specific expression among eight HLA genes by choosing the highest and lowest expressed allelic lineage with at least five samples per group (**Figure 5**). From these, we calculated the fold change (log2) between the two lineages. The highest expression differences were between pairs DPB1*02 and DPB1*03 (FC = 1.3, p = 0.009), DQA1*05 and DQA1*03 (FC = 1.3, p = 0.02), and DQB1*06 and DQB1*05 (FC = 1.3, p < 0.0001) (**Figures 5F–H**). To compare the allele-specific expression between two alleles in heterozygous allele pairs, we calculated the proportion of the HLA allele-specific expression out of the HLA gene-specific expression attributed to the less expressed allele for every heterozygote. **Table 2** shows the median and range of the calculated proportions of eight HLA genes. In addition, we divided the calculated proportions of the allele-specific expression out of the gene-specific expression into three groups. The first group, 0.01–0.20, represents the large expression differences between alleles in heterozygotes. The second group, 0.21–0.40, and the third group, 0.41–0.50, represent the groups with moderate and small expression differences, respectively. In *HLA-C, -DPB1, -DQA1*, and *-DQB1*, the mRNA expression between two alleles within an individual was the most distinct. In these genes, 10% to 14% of the heterozygous allele pairs belong to the 0.01–0.20 group (**Table 2**). In *HLA-DRB1*, 95% of the heterozygotes belong to the 0.41–0.50 group suggesting that there is very little allelic imbalance between *-DRB1* alleles within individuals.

**Table 2:** Differential allele expression between heterozygous allele pairs of eight human leukocyte antigen (HLA) genes.

Proportion of the allele-specific expression out of the gene-specific expression*					
	Median	Range	ASE 0.01–0.20 (%)	ASE 0.21–0.40 (%)	ASE 0.41–0.50 (%)
**HLA-A**	0.38	0.18–0.50	3	68	29
**HLA-B**	0.37	0.25–0.50	0	67	33
**HLA-C**	0.38	0.16–0.50	12	44	44
**HLA-DRB1**	0.47	0.37–0.50	0	5	95
**HLA-DPA1**	0.30	0.21–0.50	0	73	27
**HLA-DPB1**	0.40	0.01–0.50	13	39	47
**HLA-DQA1**	0.39	0.11–0.50	10	48	43
**HLA-DQB1**	0.33	0.11–0.50	14	58	28

*The allele-specific expression (ASE) was calculated as the proportion of the allele-specific expression out of the gene-specific expression attributed to the less expressed allele.

### HLA Haplotype Expression

**Figure 6:**
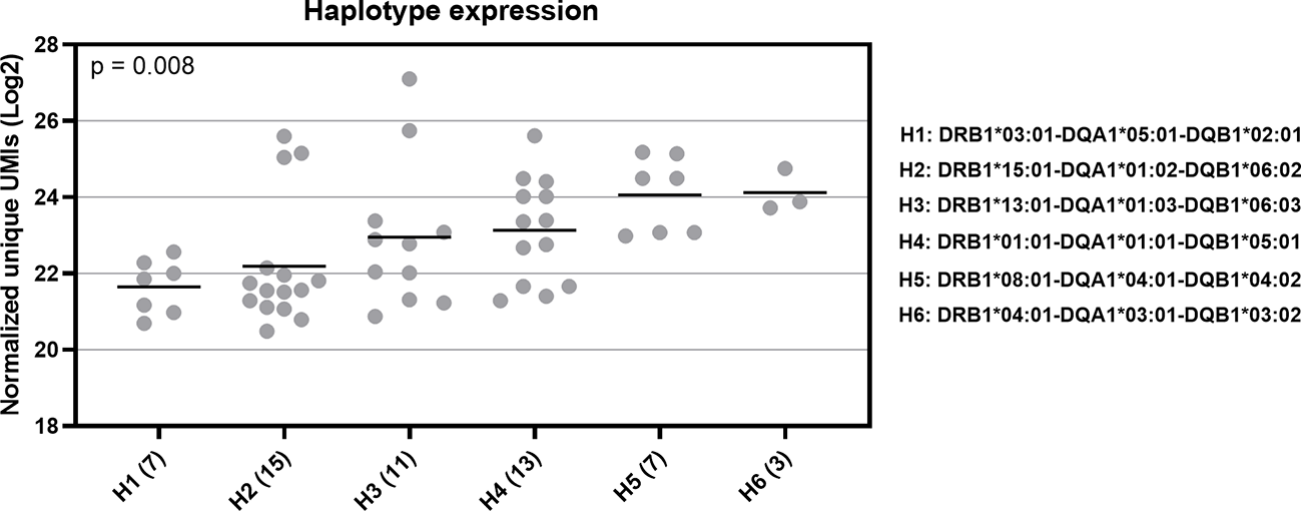
RNA expression of six common HLA haplotypes in Finnish population. Y-axis indicates the normalized UMIs (Log2) of cDNA data and X-axis indicates the codes of the five selected HLA haplotypes. The full haplotype names are marked on right side of the figure. The parenthesis following the haplotype codes indicate the number of the samples for each haplotype. Each dot refers to a UMI value and a solid bar indicates the median expression of the haplotype. Data were compared using a Kruskal-Wallis test.

We also investigated HLA haplotype expression. We selected six common haplotypes, which have a frequency of more than 5% in the Finnish population (unpublished data). These haplotypes were DRB1*03:01-DQA1*05:01-DQB1*02:01 (H1), DRB1*15:01-DQA1*01:02-DQB1*06:02 (H2), DRB1*13:01-DQA1*01:03-DQB1*06:03 (H3), DRB1*01:01-DQA1*01:01-DQB1*05:01 (H4), DRB1*08:01-DQA1*04:01-DQB1*04:02 (H5), and DRB1*04:01-DQA1*03:01-DQB1*03:02 (H6). In this comparison, we found a statistically significant difference (Kruskal-Wallis test, p = 0.008) in the RNA expression between the selected haplotypes (**Figure 6**). The H1 haplotype with the lowest expression included DQA1*05:01 and DQB1*02:01, which both had a low expression at the allotype-level based on the median expression value. In contrast, the H6 haplotype with the highest expression included alleles DQA1*03:01 and DQB1*03:02 with a high expression. Additionally, the DQ alleles of H2 were linked to the low expression and DQ alleles of H5 to either high (DQA1*04:01) or intermediate expression (DQB1*04:02). Based on these results, it seemed that *DQA1* alleles with a low expression were paired with DQB1 alleles with a low expression and *DQA1* alleles with a high expression were paired with DQB1 alleles with a high expression. However, between *DRB1* and *DQ* alleles we did not found a similar pattern. H6 with the highest expression included DRB1*04:01, which at the allotype-level had the second lowest mRNA expression. In addition, both H1 and H2 included *DRB1* alleles with an intermediate expression levels. We also compared the *DQ* haplotype expression among 50 individuals. In 95% of all of the *DQ* haplotypes included in this study, *DQA1* had lower expression than *DQB1* indicating that *DQA1* is the expression limiting molecule.

## Discussion

The data we present here demonstrates the possibility of determining both the HLA alleles and their mRNA levels using RNA-Seq methodology. Our method is applicable in various approaches related to autoimmune and transplantation genetics as well as when studying HLA expression levels in different cells and tissues. The growing body of evidence showing that HLA mRNA and surface protein expression differences may influence immune response and susceptibility to several human diseases has already catalyzed several studies using different quantitative methods to study HLA expression. The drawback with previous protein expression studies has been the lack of allele-specific monoclonal antibodies to recognize all HLA alleles with equal affinity. While qPCR has been adopted for determining the expression of HLA alleles, the focus has been mainly on HLA class I (3, 4, 6, 8). Given the high number of known HLA alleles, qPCR requires a combination of allele-specific primers to amplify different alleles of the same gene. The design of the primers can be technically challenging and time-consuming. Additionally, some studies using bulk RNA-Seq have systematically focused on the gene and especially the HLA allele-specific mRNA expression levels (32–34). However, none of the studies were based on molecule counting. By using RNA-Seq data of 50 individuals, we performed a high-throughput screen for HLA expression profiles of class I and class II alleles in peripheral blood samples.

In this study, we developed an HLA RNA-Seq method to quantify the HLA gene- and allele-specific expression based on molecule counting. Like most RNA-Seq methods, our method also involved PCR amplification steps in the library preparation protocol. To be able to amplify and process the sequencing libraries without losing information of the original molecule count, we designed a method based on STRT (54), which tags the original RNA transcripts with a molecular barcode. These barcodes, also known as UMIs, were incorporated in the 5’end of the molecules during the first strand synthesis using a TSO. We chose a 10 bp UMIs to ensure that every molecule in the original pool of RNA transcripts received a unique UMI. A ten nucleotide long UMI, which offers over 1,000,000 unique nucleotide combinations, has previously shown to be sufficient to improve the PCR duplicate removal in the data analysis steps (38). In addition to our full-length cDNA library, we also tested a targeted approach with HLA amplicons. For this, we designed HLA gene-specific primers to enrich nine HLA genes. These primers were designed to fall to the non-polymorphic area of the genes to enable efficient and cost-effective amplification of different alleles. For sequencing, we chose read lengths of 100 bp (R1) and 200 bp (R2) to ensure an adequate coverage of the HLA polymorphisms in the area encoding the peptide-binding groove in exons 2 and 3. The selected read lengths produced sufficient data for HLA typing and HLA expression quantification.

Although several tools for studying HLA mRNA expression from bulk RNA-Seq data already exist (26, 32, 34), they do not provide expression quantification with UMI counting. By using our custom pipeline, we were able accurately to determine HLA mRNA expression by counting the number of original transcripts. The comparison of allele ratios between Illumina cDNA and amplicon data showed that both approaches were able to quantify the allele-specific expression differences. However, the strength of the correlation varied between different genes. We suspect that this might be due to different efficacies of the gene-specific primers in the enrichment step. Even though the gene-specific primers were designed to fall in the non-polymorphic area of the genes, it is possible that not every allele is amplified at the same level. Because Illumina cDNA method does not include a gene-specific enrichment step, we believe it is more accurate to quantify HLA mRNA expression. However, since the allele ratios were highly concordant between the two datasets in most of the genes, the targeted approach would be a valuable option for being more cost-effective.

Our results at HLA class-level expression were consistent with previously reported findings (31, 32, 34) as HLA class I was expressed at higher levels than class II in all 50 samples. We also detected heterogeneity in the expression levels of HLA genes. Our results confirmed differential expression of HLA genes both within and between individuals. Despite a high inter-individual variation, our data showed that *HLA-B* and *HLA-C* were equally abundant on the transcript level and that their expression was about two times higher than the expression of *HLA-A*. These results are comparable with earlier gene-specific mRNA expression levels (31, 55). For class II, the order of gene-specific expression was *HLA-DRB1* > *HLA-DRA* > *HLA-DPA1* > *HLA-DPB1* > *HLA-DQB1* > *HLA-DQA1*. This imbalanced expression between HLA class II genes is in line with previous results, which have confirmed *HLA-DR* to express at higher levels compared to *HLA-DP* and *HLA-DQ* (31). However, there were also some discordances with previous findings. A study using HLA expression estimates from lymphoblastoid cell lines reported equal expression levels of *HLA-A* and *HLA-C*, while *HLA-B* showed an expression level twice as high compared to the other two class I genes (32). In contrast, another study showed the gene-level expression in the order of *HLA-B > HLA-C > HLA-A* in PBMCs (34). This same study reported that HLA-DRA was expressed at a higher level *than HLA-DRB1* and that *HLA-DPB1* was expressed at a higher level than *HLA-DPA1*. However, according to the previously reported HLA expression estimates, the gene-level expression of both *HLA-DQA1* and *HLA-DPA1* was higher than the expression of *HLA-DQB1* and *HLA-DPB1* (32). These inconsistencies between the different methods may be attributable to a number of factors, such as differences in methodology, data analysis, sample source, or population. HLA gene-specific expression has already been shown to vary between different tissues (31). Hence, it would be important to expand the expression studies to different cell types either by using bulk RNA-Seq or by selecting a single-cell approach (56).

In addition to gene-specific expression, we also investigated the HLA allotype-specific expression. Among 50 samples, we found distinct allotype-specific expression profiles. We found the largest differences between the allelic lineages in *HLA-DPB1, HLA-DQA1*, and *HLA-DQB1* indicating a strong allotype-specific expression in these genes. These results are consistent with previously reported findings, where *HLA-DQA1* and *HLA-DQB1* showed the largest differences in the inter-allelic expression (34). In the comparison of mRNA expression between two alleles in heterozygous individuals, we found the highest allelic imbalance in *HLA-C, -DPB1, -DQA1*, and *-DQB1*. In contrast, HLA-DRB1 showed almost no asymmetry in allele-specific expression within individuals suggesting that alleles in this gene are expressed in a very balanced manner. An extensive allelic imbalance for single nucleotide polymorphisms (SNPs) in the MHC has been previously described (57). However, no extreme allelic imbalance was found using HLA expression estimates (32). In HSCT, the unbalanced HLA expression between alleles in heterozygous individuals might be relevant. HLA alleles associated with low expression are suggested to be tolerated mismatches and not to increase the risk of GvHD (5, 10, 16)

Our data also demonstrated that mRNA expression differs between HLA haplotypes. This finding is concordant with earlier studies, where the diversity of MHC haplotype sequence was shown to affect the HLA gene expression (58, 59). By examining six common Finnish haplotypes, we observed distinct expression patterns. Interestingly, the haplotypes with the lowest expression (DRB1*03:01-DQA1*05:01-DQB1*02:01) and with the highest expression (DRB1*04:01-DQA1*03:01-DQB1*03:02) were both common autoimmune haplotypes. DRB1*03:01-DQA1*05:01-DQB1*02:01 has been associated with celiac disease and DRB1*04:01-DQA1*03:01-DQB1*03:02 with celiac disease (CD) and type 1 diabetes (60). Based on the distinct expression levels of these two autoimmune haplotypes in PBMCs, we could argue that the expression level itself might not be a risk factor. However, more information on the expression of these predisposing haplotypes between patients affected by the disease and healthy controls is needed.

It is of note that we analyzed the peripheral blood samples without any quantification of their cellular contents, and it is not clear how much variation in immune cell numbers affects the inter-individual results. The intra-allelic variation we see in our results might partially be due to the differences in cell subpopulation distributions, and hence the results should be interpreted with caution. To exclude any variation originating from differences in the sample material, we recommend that further studies of HLA gene- and allele-specific expression with known cellular composition and ratios are conducted to explore the effect of different blood cell types on HLA class I, and II expression. We are also aware of other factors, both genetic and environmental, which might alter HLA expression. The impact of different factors such as age, medication, infection status, activation status, and history on HLA expression should be assessed more closely in controlled studies in the future. Furthermore, in this study, we did not investigate the effect of any regulatory elements on HLA mRNA expression. However, since several polymorphisms located both in the 5’-, and 3’- untranslated region (UTR) have been previously associated with varying HLA expression levels (4, 61, 62), it would be important to further investigate the non-exon regions to locate additional SNPs regulating HLA expression.

In this study, we have developed a novel method for exploring the complexity of HLA gene- and allele-level expression from 5’end bulk RNA-Seq data using UMIs. Increasing information on different factors affecting the outcome of the HSCT may cause challenges for identifying suitable donors meeting all required criteria. Therefore, our aim is to propose a tool to explore the differential HLA allele expression profiles. In the future, expression screening of HLA alleles could help in the discovery of possible permissive mismatches. Such tolerated mismatches could be beneficial in avoiding high-risk transplantations making HSCTs safer when no matched donor is available. Since several research and clinical HLA laboratories have already adopted NGS in HLA typing, the leap from DNA sequencing to RNA-Seq enabling both the HLA typing and expression quantification could be possible in the future. This would change the nature of HLA research from qualitative to a quantitative field of science.

## Conclusions

In summary, our HLA RNA-Seq method with UMIs was able to quantify mRNA expression at the gene- and allele-level. We identified expression differences between HLA genes and HLA allelic lineages. Identification of these allele-specific expression differences could be important for future HLA disease association studies and in HSCT to find permissive mismatches when no matched donor is available. We note that the method presented in this study can be applied to quantify the mRNA expression of HLA alleles in various cells and tissues.

## Data Availability Statement

The datasets presented in this study can be found in online repositories. The names of the repository/repositories and accession number(s) can be found below: EGA European Genome-Phenome Archive [accession: EGAS00001004931].

## Author Contributions

TJ, PS, and JP designed the study. TJ managed all the samples and prepared the sequencing libraries. SK managed Luminex HLA typing. TJ and DY designed the data analysis pipeline. TJ and DY performed the data analyses and TJ interpreted the data. TJ, PS, and JP interpreted the results. TJ and DY wrote the manuscript. All authors contributed to the article and approved the submitted version.

## Funding

This work was financially supported by grants from Clinical Research Funding (EVO/VTR), the Academy of Finland, Tekes (the Finnish Funding Agency for Technology and Innovation), the Väre Foundation for Pediatric Cancer Research, and the Finnish Concordia Fund. The funding bodies had no role in the study design, sample collection, analysis and interpretation of data and in writing the manuscript.

## Conflict of Interest

The authors declare that the research was conducted in the absence of any commercial or financial relationships that could be construed as a potential conflict of interest.
