# Supplementary material for: HLA RNA Sequencing With Unique Molecular Identifiers Reveals High Allele-Specific Variability in mRNA Expression
Source: Front Immunol. 2021 Feb 25;12:629059. doi: 10.3389/fimmu.2021.629059 (PMC7949471; doi:10.3389/fimmu.2021.629059)
Supplement: Supplementary file 1 [file DataSheet_1.docx]

**Method S1. HLA genotyping**

HLA typing of HLA-A, -B, -C, -DRA, -DRB1, -DQA1, -DQB1, -DPA1 and -DPB1 alleles for Illumina amplicon data was done using three different typing softwares: Omixon Explore (v1.2.0, Omixon), HLAProfiler [1], and an in-house HLA-typing tool. Allele-level typing results from these three softwares were run through a majority voting pipeline [2] producing an ensemble prediction of HLA alleles. For validation of HLA typing, genomic DNA was extracted from PBMC samples by QiaSymphony SP automat (Qiagen) by following the manufacturer’s protocol. The concentration and purity of DNA was measured by NanoDrop ND-1000 (Thermo Fisher Scientific). The reverse SSOP-Luminex technology (Labtype, One Lambda) was used for HLA typing of HLA-A, -B, -C, -DRB1, -DRB3, -DRB4, -DRB5, -DQA1, -DQB1, -DPA1, and -DPB1. The results were analyzed with the HLA-Fusion software (v.3.2.0-HF1, One Lambda). The concordance rate of the genotyping results between Illumina data and Luminex data was calculated, In case of a disconcordant result or a rare allele in the ensemble genotyping results, we checked the allele frequency in the Finnish population using a Finnish cohort study [3]. If the allele assigned by ensemble method was not present in either of the cohorts, it was corrected to a more common allele. This was either the first allele in the list of alleles by Luminex or in some cases the most frequent allele option according to the cohort [3].

**References**

1. Buchkovich ML, Brown CC, Robasky K, Chai S, Westfall S, Vincent BG, et al. HLAProfiler utilizes k-mer profiles to improve HLA calling accuracy for rare and common alleles in RNA-seq data. Genome Med. 2017;9(1):1–15.

2. Larjo A, Eveleigh R, Kilpeläinen E, Kwan T, Pastinen T. Accuracy of Programs for the Determination of Human Leukocyte Antigen Alleles from Next-Generation Sequencing Data. 2017;8(December):1–9.

3. Haimila K, Peräsaari J, Linjama T, Koskela S, Saarinenl T, Lauronen J, et al. HLA antigen, allele and haplotype frequencies and their use in virtual panel reactive antigen calculations in the Finnish population. Tissue Antigens. 2013;81(1):35–43.
