# Supplementary material for: HLA RNA Sequencing With Unique Molecular Identifiers Reveals High Allele-Specific Variability in mRNA Expression
Source: Front Immunol. 2021 Feb 25;12:629059. doi: 10.3389/fimmu.2021.629059 (PMC7949471; doi:10.3389/fimmu.2021.629059)
Supplement: Supplementary file 2 [file DataSheet_2.docx]

**Method S2. Comparison of gene and allele-specific expression between HLA RNA sequencing with UMIs and qPCR.**

Five RNA samples were diluted to 20 ng/µl and reverse transcribed with the iScript cDNA synthesis kit (Bio-Rad). cDNA was quantified relative to B2M using iQ™ SYBR® Green supermix kit (Bio-Rad) and CFX96 Touch System (Bio-Rad). For B2M, the forward and reverse primers (IDT) were 5’TGCTGTCTCCATGTTTGATGTATCT3’ and 5’TCTCTGCTCCCCACCTCTAAGT3’, respectively. Both the HLA-C gene-specific primer and the allele-specific primers have been previously described [1,2]. The PCR efficiency was tested using cDNA dilution series and by comparing the slopes (Figure S1). Allele-positive and -negative samples were used to control the specificity of the primers. All reactions were performed in triplicate. The gene-specific expression was analyzed using the$2^{-\Delta\Delta C_{T}}$ method [3] and the allele-specific using the Pfaffl ratio method [4] to minimize the variation of PCR efficiencies of primers.

**Figure S1**

**Supplementary Figure 1. cDNA dilution curves of the primers.** Y-axis indicates the Cq-value and X-axis indicates the cDNA dilution series. The legend key indicates the colors of the different primers.

**Figure S2**

**
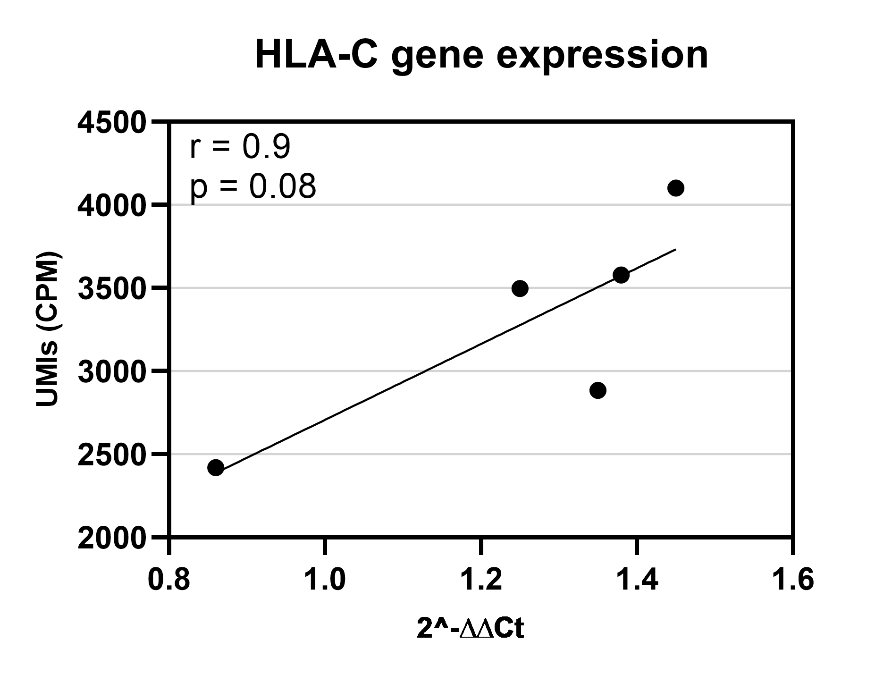
**

**Supplementary Figure 2. Comparison of the HLA-C gene-specific expression between HLA RNA sequencing using UMIs and qPCR.** Y-axis indicates the UMIs of HLA RNA sequencing method and X-axis indicates the mRNA expression values from qPCR ($2^{-\Delta\Delta C_{T}})$. A Spearman correlation was used to analyze the data.

**Figure S3**

C*07:01

C*07:01

C*07:01

C*07:01

C*07:01

C*07:01

C*07:01

C*07:01

C*07:01

C*07:01

C*04:01

C*06:02

C*06:02

C*06:02

C*06:02

C*06:02

C*06:02

C*06:02

C*06:02

**Supplementary Figure 3. Comparison of the allele-specific** **mRNA expression patterns of five samples between HLA RNA sequencing and qPCR.** The left Y-axis indicates the mRNA expression values from qPCR (Pfaffl ratio) and the right Y-axis UMIs of HLA RNA sequencing method. X-axis indicates the sample names. The legend key shows the marker information. The allele names are shown next to the markers.

**References**

1. Bettens F, Brunet L, Tiercy J-M. High-allelic variability in HLA-C mRNA expression: association with HLA-extended haplotypes. Genes Immun. 2014;15(10):176–81.

2. Bunce M, O’Neill CM, Barnardo MC, Krausa P, Browning MJ, Morris PJ, et al. Phototyping: comprehensive DNA typing for HLA-A, B, C, DRB1, DRB3, DRB4, DRB5 & DQB1 by PCR with 144 primer mixes utilizing sequence-specific primers (PCR-SSP). Tissue Antigens. 1995 Nov;46(5):355–67.

3. Livak KJ, Schmittgen TD. Analysis of relative gene expression data using real-time quantitative PCR and the 2-ΔΔCT method. Methods. 2001;25(4):402–8.

4. Pfaffl MW. A new mathematical model for relative quantification in real-time RT-PCR. Nucleic Acids Res [Internet]. 2001 May 1;29(9):e45–e45. Available from: https://pubmed.ncbi.nlm.nih.gov/11328886
