# Supplementary material for: HLA RNA Sequencing With Unique Molecular Identifiers Reveals High Allele-Specific Variability in mRNA Expression
Source: Front Immunol. 2021 Feb 25;12:629059. doi: 10.3389/fimmu.2021.629059 (PMC7949471; doi:10.3389/fimmu.2021.629059)
Supplement: Supplementary file 3 [file DataSheet_3.docx]

**Figure S4.**


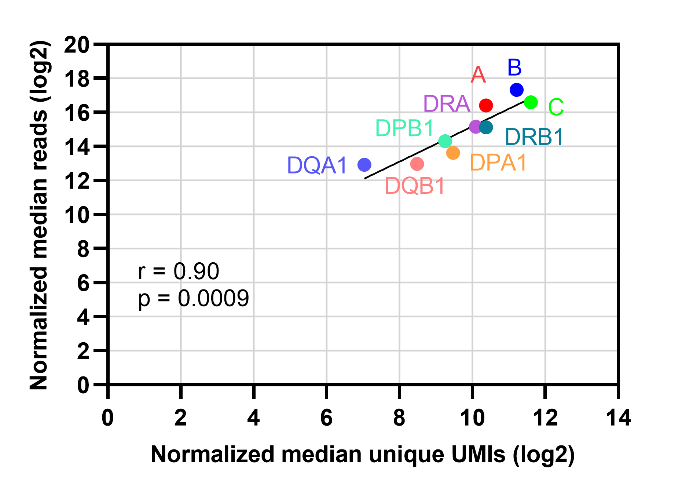


**Supplementary Figure 4. Comparison of the expression of nine HLA genes between HLA RNA sequencing using UMIs and RNA capture and Illumina RNA sequencing method.** X-axis indicates the normalized median unique UMIs (log2) of HLA RNA sequencing method using UMIs and Y-axis indicates the normalized median reads (log2) of RNA of the capture and Illumina RNA sequencing method. A Pearson correlation was used to analyze the data.
