# Supplementary material for: HLA RNA Sequencing With Unique Molecular Identifiers Reveals High Allele-Specific Variability in mRNA Expression
Source: Front Immunol. 2021 Feb 25;12:629059. doi: 10.3389/fimmu.2021.629059 (PMC7949471; doi:10.3389/fimmu.2021.629059)
Supplement: Supplementary file 4 [file DataSheet_4.zip › Supplementary Table 1.DOCX]

| Table S1. List of primer sequences. | |  |
| --- | --- | --- |
|  | |  |
|  | |  |
| Primer | **Primer sequence** | |
| STRT-V3-T30-VN oligo | 5’-biotin-TTAAGCAGTGGTATCAACGCAGAGTCGAC30VN-3’ | |
| RNA-TSO 10bp UMI | 5’-biotin-CAGUGGUAUCAACGCAGAGUNNNNNNNNNNrGrGrG-3’ | |
| ImSTRT-TSO-PCR | 5-’CAGTGGTATCAACGCAGAGT-3’ | |
|  |  | |
| Gene-specific primers |  | |
| HLA-ABC-specific universal reverse | 5'-GTGACTGGAGTTCAGACGTGTGCTCTTCCGAT**CTATCAGAGCCCTGGGCACTGT-3’** | |
| HLA-G-specific universal reverse | 5'-GTGACTGGAGTTCAGACGTGTGCTCTTCCGATCT**GGCTCTCCTTTGTTCAGCCACA-3’** | |
| DRA-specific universal reverse | 5'-GTGACTGGAGTTCAGACGTGTGCTCTTCCGATCT**CTTGGAGCATCAAACTCCCAGTG-3’** | |
| DRB-specific universal reverse | 5'-GTGACTGGAGTTCAGACGTGTGCTCTTCCGATCT**GTGCTGCAGGGGCTGGGTCT-3’** | |
| DPA1-specific universal reverse | 5'-GTGACTGGAGTTCAGACGTGTGCTCTTCCGATCT**AGAGGCTCTCAGCGACACCCTC-3’** | |
| DPB1-specific universal reverse | 5'-GTGACTGGAGTTCAGACGTGTGCTCTTCCGATCT**GTCACGTGGCAGACAAGCAGGTTG-3’** | |
| DQA1-specific universal reverse | 5'-GTGACTGGAGTTCAGACGTGTGCTCTTCCGATCT**CAGTGCTCCACCTTGCAGTCATAA-3’** | |
| DQB1-specific universal reverse | 5'-GTGACTGGAGTTCAGACGTGTGCTCTTCCGATCT**AGGGCCTCTGTCCTGGATGGGG-3’** | |
|  |  | |

| **Index primer** | | **Primer sequence** | |
| --- | --- | --- | --- |
| P7 Adaptor **index i7** | | | |
| Nextera_N701 | | CAA GCA GAA GAC GGC ATA CGA GAT **TCG CCT TA**G TCT CGT GGG CTC GG | |
| Nextera_N702 | | CAA GCA GAA GAC GGC ATA CGA GAT **CTA GTA CG**G TCT CGT GGG CTC GG | |
| Nextera_N703 | | CAA GCA GAA GAC GGC ATA CGA GAT **TTC TGC CT**G TCT CGT GGG CTC GG | |
| Nextera_N704 | | CAA GCA GAA GAC GGC ATA CGA GAT **GCT CAG GA**G TCT CGT GGG CTC GG | |
| Nextera_N705 | | CAA GCA GAA GAC GGC ATA CGA GAT **AGG AGT CC**G TCT CGT GGG CTC GG | |
| Nextera_N706 | | CAA GCA GAA GAC GGC ATA CGA GAT **CAT GCC TA**G TCT CGT GGG CTC GG | |
| Nextera_N707 | | CAA GCA GAA GAC GGC ATA CGA GAT **GTA GAG AG**G TCT CGT GGG CTC GG | |
| Nextera_N710 | | CAA GCA GAA GAC GGC ATA CGA GAT **CAG CCT CG**G TCT CGT GGG CTC GG | |
| Nextera_N711 | | CAA GCA GAA GAC GGC ATA CGA GAT **TGC CTC TT**G TCT CGT GGG CTC GG | |
| Nextera_N712 | | CAA GCA GAA GAC GGC ATA CGA GAT **TCC TCT AC**G TCT CGT GGG CTC GG | |
| Nextera_N714 | | CAA GCA GAA GAC GGC ATA CGA GAT **TCA TGA GC**G TCT CGT GGG CTC GG | |
| Nextera_N715 | | CAA GCA GAA GAC GGC ATA CGA GAT **CCT GAG AT**G TCT CGT GGG CTC GG | |
| Nextera_N716 | | CAA GCA GAA GAC GGC ATA CGA GAT **TAG CGA GT**G TCT CGT GGG CTC GG | |
| Nextera_N718 | | CAA GCA GAA GAC GGC ATA CGA GAT **GTA GCT CC**G TCT CGT GGG CTC GG | |
| Nextera_N719 | | CAA GCA GAA GAC GGC ATA CGA GAT **TAC TAC GC**G TCT CGT GGG CTC GG | |
| Nextera_N720 | | CAA GCA GAA GAC GGC ATA CGA GAT **AGG CTC CG**G TCT CGT GGG CTC GG | |
| Nextera_N721 | | CAA GCA GAA GAC GGC ATA CGA GAT **GCA GCG TA**G TCT CGT GGG CTC GG | |
| Nextera_N722 | | CAA GCA GAA GAC GGC ATA CGA GAT **CTG CGC AT**G TCT CGT GGG CTC GG | |
| Nextera_N723 | | CAA GCA GAA GAC GGC ATA CGA GAT **GAG CGC TA**G TCT CGT GGG CTC GG | |
| Nextera_N724 | | CAA GCA GAA GAC GGC ATA CGA GAT **CGC TCA GT**G TCT CGT GGG CTC GG | |
| Nextera_N726 | | CAA GCA GAA GAC GGC ATA CGA GAT **GTC TTA GG**G TCT CGT GGG CTC GG | |
| Nextera_N727 | | CAA GCA GAA GAC GGC ATA CGA GAT **ACT GAT CG**G TCT CGT GGG CTC GG | |
| Nextera_N728 | | CAA GCA GAA GAC GGC ATA CGA GAT **TAG CTG CA**G TCT CGT GGG CTC GG | |
| Nextera_N729 | | CAA GCA GAA GAC GGC ATA CGA GAT **GAC GTC GA**G TCT CGT GGG CTC GG | |
|  | |  | |
|  | | P5 Adaptor **index i5** custom TSO sequence | |
| ImSTRT-D501L | | 5’AATGATACGGCGACCACCGAGATCTACAC**TATAGCCT**ACGCAGTGCTCAGTGGTATCAACGCAGAGT | |
| ImSTRT-D502L | | 5’AATGATACGGCGACCACCGAGATCTACAC**ATAGAGGC**ACGCAGTGCTCAGTGGTATCAACGCAGAGT | |
| ImSTRT-D503L | | 5’AATGATACGGCGACCACCGAGATCTACAC**CCTATCCT**ACGCAGTGCTCAGTGGTATCAACGCAGAGT | |
| ImSTRT-D504L | | 5’AATGATACGGCGACCACCGAGATCTACAC**GGCTCTGA**ACGCAGTGCTCAGTGGTATCAACGCAGAGT | |
| ImSTRT-D505L | | 5’AATGATACGGCGACCACCGAGATCTACAC**AGGCGAAGA**CGCAGTGCTCAGTGGTATCAACGCAGAGT | |
| ImSTRT-D506L | | 5’AATGATACGGCGACCACCGAGATCTACAC**TAATCTTA**ACGCAGTGCTCAGTGGTATCAACGCAGAGT | |
| ImSTRT-D507L | | 5’AATGATACGGCGACCACCGAGATCTACAC**CAGGACGT**ACGCAGTGCTCAGTGGTATCAACGCAGAGT | |
| ImSTRT-D508L | | 5’AATGATACGGCGACCACCGAGATCTACAC**GTACTGAC**ACGCAGTGCTCAGTGGTATCAACGCAGAGT | |
|  | |  | |
|  |  | |  |
| Primers used in RT HLA amplicon enrichment and Illumina Nextera XT library preparation for multiplexing HLA amplicon and cDNA libraries. Nextera_N7XX primers include Illumina P7 adaptor and i7 index. ImSTRT-D50XL primers include Illumina P5 adaptor, i5 index, and the custom TSO. | | |  |
