# Supplementary material for: HLA RNA Sequencing With Unique Molecular Identifiers Reveals High Allele-Specific Variability in mRNA Expression
Source: Front Immunol. 2021 Feb 25;12:629059. doi: 10.3389/fimmu.2021.629059 (PMC7949471; doi:10.3389/fimmu.2021.629059)
Supplement: Supplementary file 4 [file DataSheet_4.zip › Supplementary Table 2.DOCX]

| **Table S2. The number of different HLA alleles of 50 individuals at 2-field genotyping resolution** | | | | |  |
| --- | --- | --- | --- | --- | --- |
| **HLA class I** | |  |  |  |  |
| **HLA-A alleles** | **Number of alleles** | **HLA-B alleles** | **Number of alleles** | **HLA-C alleles** | **Number of alleles** |
| A*01:01 | 8 | B*07:02 | 19 | C*01:02 | 4 |
| A*02:01 | 26 | B*08:01 | 7 | C*02:02 | 3 |
| A*03:01 | 30 | B*13:02 | 6 | C*03:03 | 8 |
| A*03:03 | 1 | B*14:02 | 1 | C*03:04 | 9 |
| A*11:01 | 2 | B*15:01 | 10 | C*04:01 | 20 |
| A*24:02 | 18 | B*18:01 | 6 | C*05:01 | 4 |
| A*25:01 | 2 | B*27:02 | 6 | C*06:02 | 10 |
| A*26:01 | 1 | B*35:01 | 14 | C*07:01 | 16 |
| A*29:01 | 1 | B*35:03 | 2 | C*07:02 | 15 |
| A*29:02 | 1 | B*35:05 | 1 | C*07:04 | 1 |
| A*31:01 | 2 | B*35:08 | 1 | C*08:02 | 1 |
| A*32:01 | 4 | B*37:01 | 1 | C*12:03 | 3 |
| A*33:01 | 1 | B*39:01 | 4 | C*15:02 | 5 |
| A*68:01 | 3 | B*39:06 | 1 | C*17:01 | 738 |
|  |  | B*40:01 | 5 |  |  |
|  |  | B*41:01 | 1 |  |  |
|  |  | B*44:02 | 4 |  |  |
|  |  | B*44:27 | 1 |  |  |
|  |  | B*47:01 | 1 |  |  |
|  |  | B*49:01 | 1 |  |  |
|  |  | B*51:01 | 4 |  |  |
|  |  | B*55:01 | 2 |  |  |
|  |  | B*56:01 | 1 |  |  |
|  |  | B*57:01 | 1 |  |  |
|  |  |  |  |  |  |
|  |  |  |  |  |  |
|  |  |  |  |  |  |
|  |  |  |  |  |  |
|  |  |  |  |  |  |
| **HLA class II** |  |  |  |  |  |
| **HLA-DRA alleles** | **Number of alleles** | **HLA-DRB1 alleles** | **Number of alleles** | **HLA-DPA1 alleles** | **Number of alleles** |
| DRA*01:01 | 68 | DRB1*01:01 | 16 | DPA1*01:03 | 88 |
| DRA*01:02 | 32 | DRB1*01:02 | 1 | DPA1*01:04 | 2 |
|  |  | DRB1*03:01 | 7 | DPA1*02:01 | 6 |
|  |  | DRB1*04:01 | 6 | DPA1*02:02 | 4 |
|  |  | DRB1*04:03 | 2 |  |  |
|  |  | DRB1*04:04 | 2 |  |  |
|  |  | DRB1*04:07 | 1 |  |  |
|  |  | DRB1*07:01 | 8 |  |  |
|  |  | DRB1*08:01 | 10 |  |  |
|  |  | DRB1*09:01 | 2 |  |  |
|  |  | DRB1*10:01 | 3 |  |  |
|  |  | DRB1*11:01 | 1 |  |  |
|  |  | DRB1*12:01 | 2 |  |  |
|  |  | DRB1*13:01 | 12 |  |  |
|  |  | DRB1*13:02 | 5 |  |  |
|  |  | DRB1*14:54 | 1 |  |  |
|  |  | DRB1*15:01 | 17 |  |  |
|  |  | DRB1*16:01 | 4 |  |  |
| **HLA-DPB1 alleles** | **Number of alleles** | **HLA-DQA1 alleles** | **Number of alleles** | **HLA-DQB1 alleles** | **Number of alleles** |
| DPB1*01:01 | 3 | DQA1*01:01 | 17 | DQB1*02:01 | 9 |
| DPB1*02:01 | 11 | DQA1*01:02 | 26 | DQB1*02:02 | 3 |
| DPB1*03:01 | 14 | DQA1*01:03 | 12 | DQB1*03:01 | 7 |
| DPB1*04:01 | 41 | DQA1*01:04 | 1 | DQB1*03:03 | 4 |
| DPB1*04:02 | 19 | DQA1*01:05 | 3 | DQB1*04:02 | 9 |
| DPB1*05:01 | 4 | DQA1*02:01 | 9 | DQB1*05:01 | 20 |
| DPB1*14:01 | 3 | DQA1*03:01 | 9 | DQB1*05:02 | 4 |
| DPB1*15:01 | 3 | DQA1*03:02 | 1 | DQB1*05:03 | 1 |
| DPB1*20:01 | 3 | DQA1*03:03 | 4 | DQB1*06:02 | 17 |
| DPB1*23:01 | 1 | DQA1*04:01 | 9 | DQB1*06:03 | 12 |
|  |  | DQA1*05:01 | 9 | DQB1*06:04 | 5 |
