# Supplementary material for: HLA RNA Sequencing With Unique Molecular Identifiers Reveals High Allele-Specific Variability in mRNA Expression
Source: Front Immunol. 2021 Feb 25;12:629059. doi: 10.3389/fimmu.2021.629059 (PMC7949471; doi:10.3389/fimmu.2021.629059)
Supplement: Supplementary file 4 [file DataSheet_4.zip › Supplementary Table 5.DOCX]

**Table S5. Statistical difference between the expression levels of HLA genes**

| **Dunn's multiple comparisons test** | **Significant** | **Adjusted P Value** |
| --- | --- | --- |
| C vs. B | No | >0,9999 |
| C vs. DRB1 | Yes | 0,0003 |
| C vs. A | Yes | 0,0008 |
| C vs. DRA | Yes | <0,0001 |
| C vs. DPA1 | Yes | <0,0001 |
| C vs. DPB1 | Yes | <0,0001 |
| C vs. DQB1 | Yes | <0,0001 |
| C vs. DQA1 | Yes | <0,0001 |
| B vs. DRB1 | Yes | 0,0373 |
| B vs. A | No | 0,0717 |
| B vs. DRA | Yes | 0,001 |
| B vs. DPA1 | Yes | <0,0001 |
| B vs. DPB1 | Yes | <0,0001 |
| B vs. DQB1 | Yes | <0,0001 |
| B vs. DQA1 | Yes | <0,0001 |
| DRB1 vs. A | No | >0,9999 |
| DRB1 vs. DRA | No | >0,9999 |
| DRB1 vs. DPA1 | Yes | 0,0043 |
| DRB1 vs. DPB1 | Yes | <0,0001 |
| DRB1 vs. DQB1 | Yes | <0,0001 |
| DRB1 vs. DQA1 | Yes | <0,0001 |
| A vs. DRA | No | >0,9999 |
| A vs. DPA1 | Yes | 0,0023 |
| A vs. DPB1 | Yes | <0,0001 |
| A vs. DQB1 | Yes | <0,0001 |
| A vs. DQA1 | Yes | <0,0001 |
| DRA vs. DPA1 | No | 0,1192 |
| DRA vs. DPB1 | Yes | 0,001 |
| DRA vs. DQB1 | Yes | <0,0001 |
| DRA vs. DQA1 | Yes | <0,0001 |
| DPA1 vs. DPB1 | No | >0,9999 |
| DPA1 vs. DQB1 | No | 0,1165 |
| DPA1 vs. DQA1 | Yes | <0,0001 |
| DPB1 vs. DQB1 | No | >0,9999 |
| DPB1 vs. DQA1 | Yes | 0,0015 |
| DQB1 vs. DQA1 | No | 0,5305 |
